# Supplementary material for: Optimizing laboratory-based surveillance networks for monitoring multi-genotype or multi-serotype infections
Source: PLoS Comput Biol. 2022 Sep 27;18(9):e1010575. doi: 10.1371/journal.pcbi.1010575 (PMC9543988; doi:10.1371/journal.pcbi.1010575)
Supplement: S1 Text — (DOCX) [file pcbi.1010575.s001.docx]

## S1 Text. Epidemiological parameter estimates by the disease system model

Figure ST1.1 shows 100 realizations of the serotype-specific incidence rates. It illustrates that, at the province level, the incidence rate caused by non-CV-A16 and non-EV-A71 serotypes increased steadily over the surveillance period, while CV-A16 and EV-A71 alternate as the dominant known serotype, although the exact patterns vary from prefecture to prefecture (Figure ST1.2). These findings are consistent with the results of previous studies in the region [1], and similar patterns have been observed in other parts of China [2-4]. In accordance with previous studies [5, 6], we also found that EV-A71 had the highest—and CV-A16 the lowest—probability of causing severe disease. EV-A71, CV-A16, and other enterovirus were estimated to have probabilities of 0.020 (95% CI: 0.019, 0.021), 0.0012 (95% credible interval (CI): 0.0010, 0.0014), and 0.0072 (95% CI: 0.0067, 0.0077) of causing severe cases, respectively (Figure ST1.3).


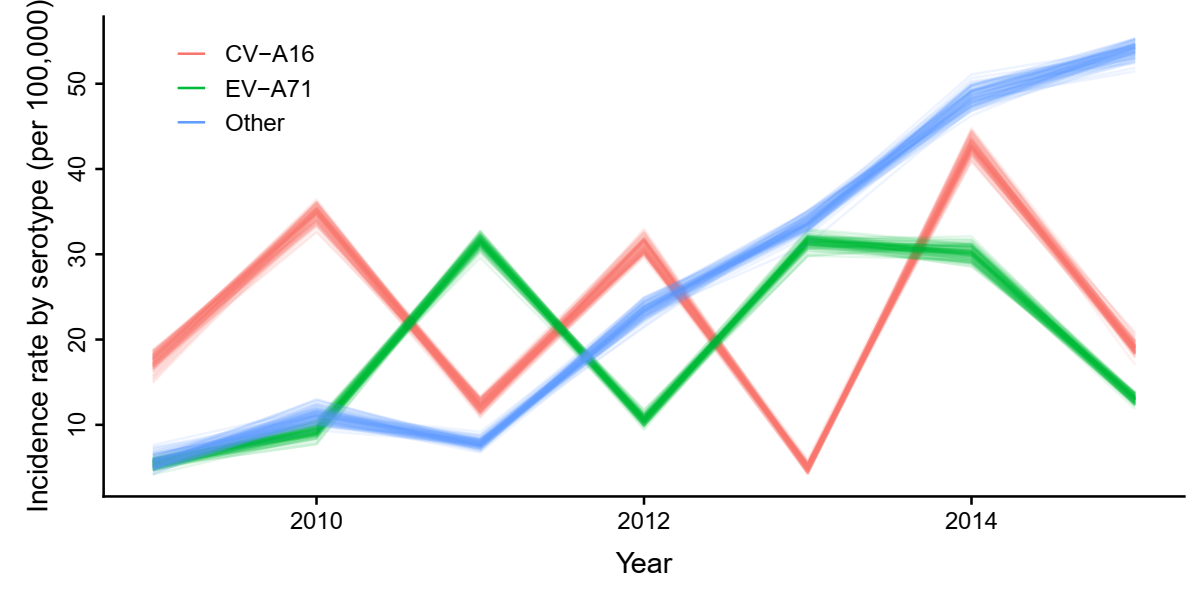


**Figure ST1.1**. Serotype-specific incidence rate estimated from 100 realizations of the disease system model.


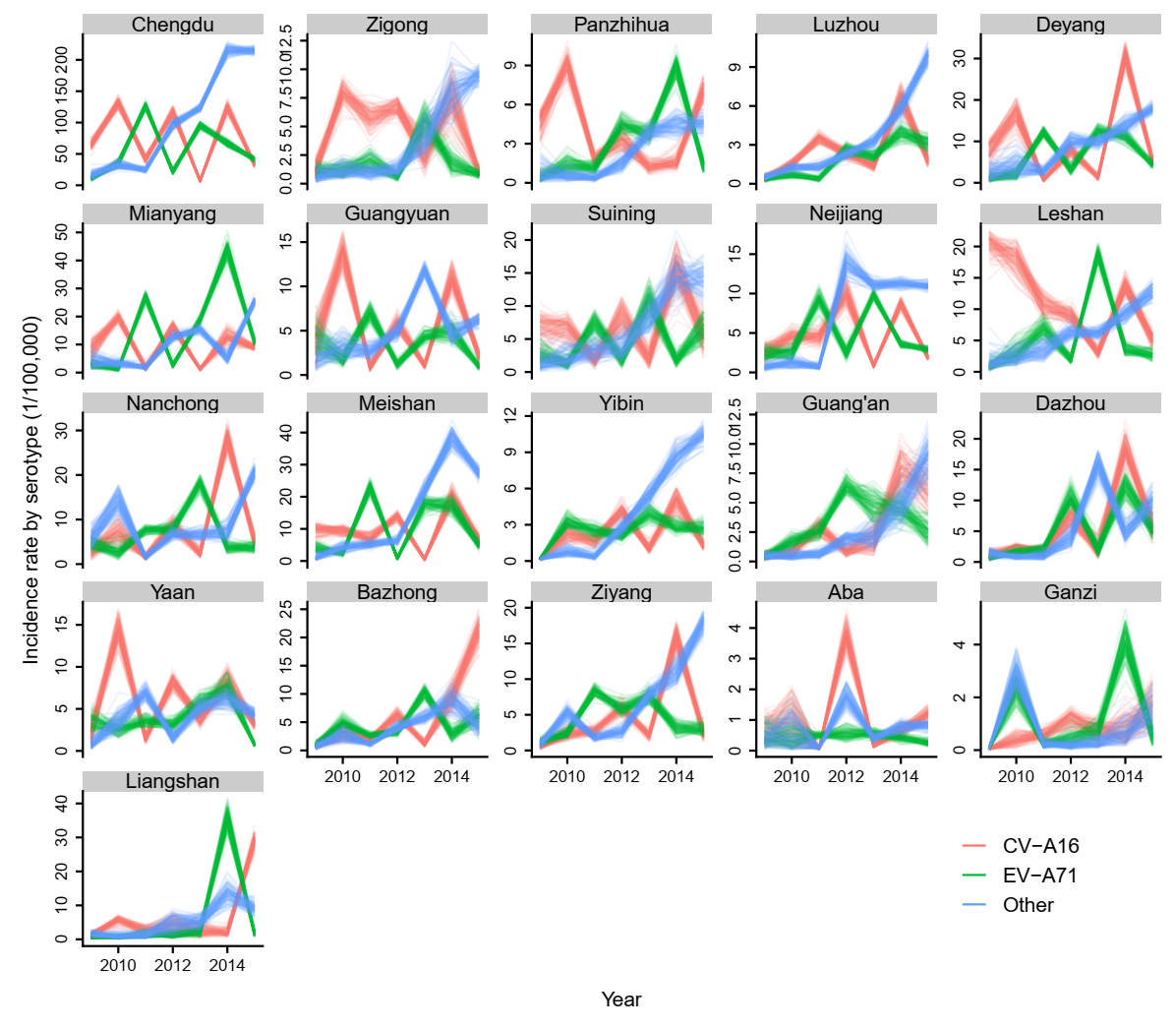


**Figure ST1.2.** Serotype-specific incidence rate estimated from 100 realizations of the disease system model for each prefecture.


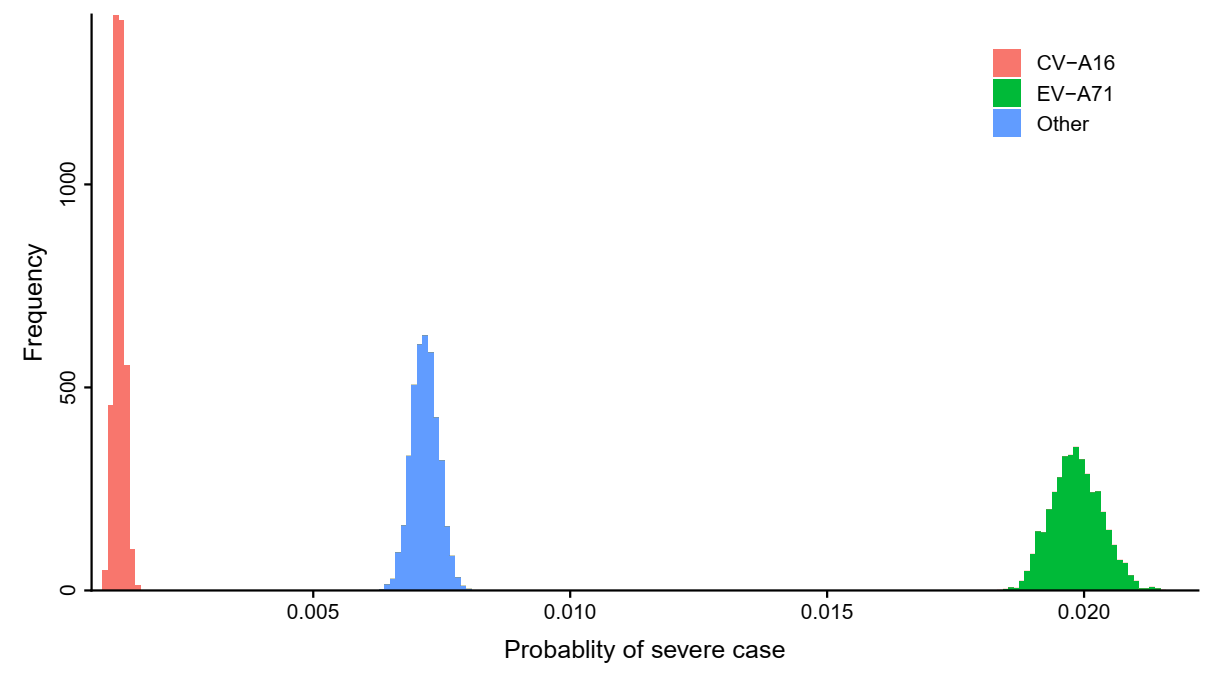


**Figure ST1.3.** Posterior distribution of the probability of each serotype causing severe case estimated by the disease system model.

**References for Supplementary Information**

1. Head JR, Collender PA, Lewnard JA, Skaff NK, Li L, Cheng Q, et al. Early Evidence of Inactivated Enterovirus 71 Vaccine Impact Against Hand, Foot, and Mouth Disease in a Major Center of Ongoing Transmission in China, 2011–2018: A Longitudinal Surveillance Study. Clinical Infectious Diseases. 2019.

2. Xu Y, Zheng Y, Shi W, Guan L, Yu P, Xu J, et al. Pathogenic characteristics of hand, foot and mouth disease in Shaanxi Province, China, 2010–2016. Scientific Reports. 2020;10.

3. Li Y, Bao H, Zhang X, Zhai M, Bao X, Wang D, et al. Epidemiological and genetic analysis concerning the non‐enterovirus 71 and non‐coxsackievirus A16 causative agents related to hand, foot and mouth disease in Anyang city, Henan Province, China, from 2011 to 2015. Journal of medical virology. 2017;89(10):1749-58.

4. Wang J, Zhou J, Xie G, Zheng S, Lou B, Chen Y, et al. The Epidemiological and Clinical Characteristics of Hand, Foot, and Mouth Disease in Hangzhou, China, 2016 to 2018. Clinical Pediatrics. 2020;59(7):656-62.

5. Tang X, Yang Y, Yu H-J, Liao Q-H, Bliznyuk N. A Spatio-Temporal Modeling Framework for Surveillance Data of Multiple Infectious Pathogens With Small Laboratory Validation Sets. Journal of the American Statistical Association. 2019;114(528):1561-73.

6. Lei X, Cui S, Zhao Z, Wang J. Etiology, pathogenesis, antivirals and vaccines of hand, foot, and mouth disease. National Science Review. 2015;2(3):268-84.
