# Supplementary figures and images for: Optimizing laboratory-based surveillance networks for monitoring multi-genotype or multi-serotype infections
Source: PLoS Comput Biol. 2022 Sep 27;18(9):e1010575. doi: 10.1371/journal.pcbi.1010575 (PMC9543988; doi:10.1371/journal.pcbi.1010575)

**(A) All clinical cases**

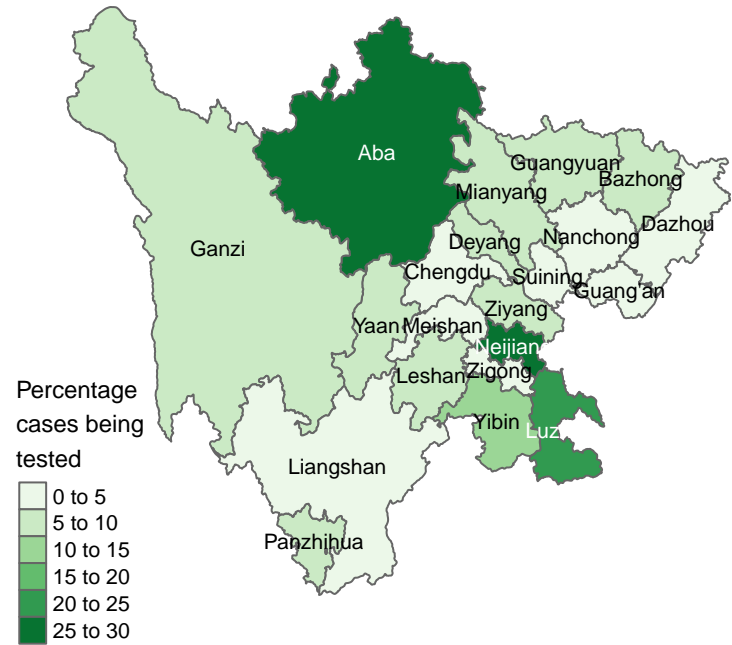

**(B) Mild cases**

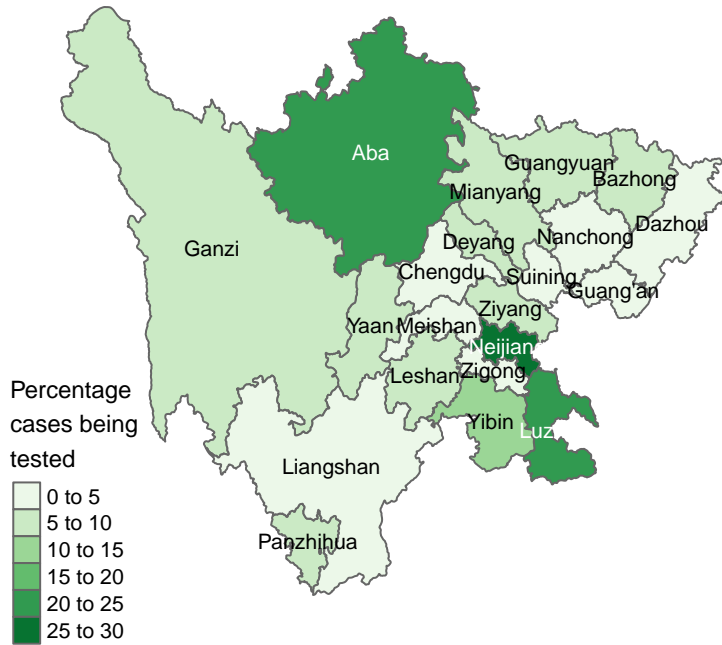

**(C) Severe cases**

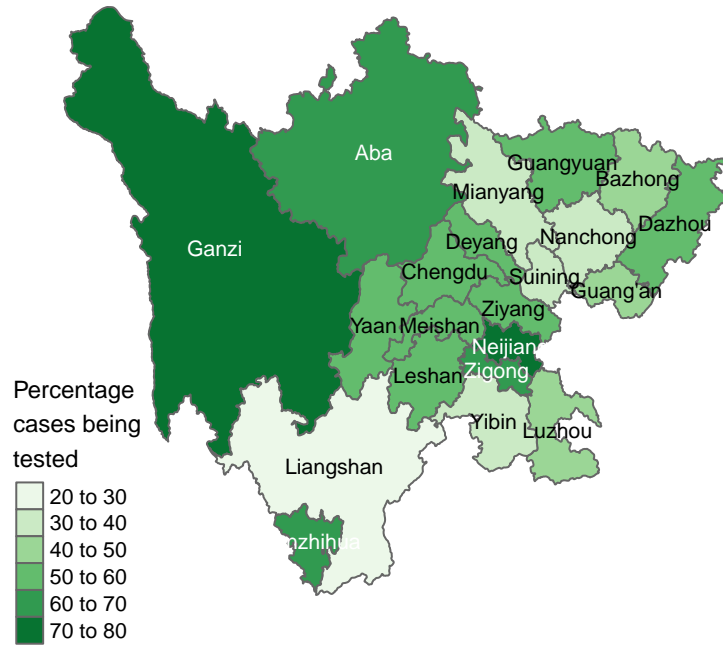

Supplement: S1 Fig — Annual mean percentage of cases being tested for (A) all clinical HFMD cases, (B) mild cases, and (C) severe cases between 2009–2015. The boundaries of the prefectures were obtained from https://gadm.org/download_country.html. (PDF) [file pcbi.1010575.s002.pdf]

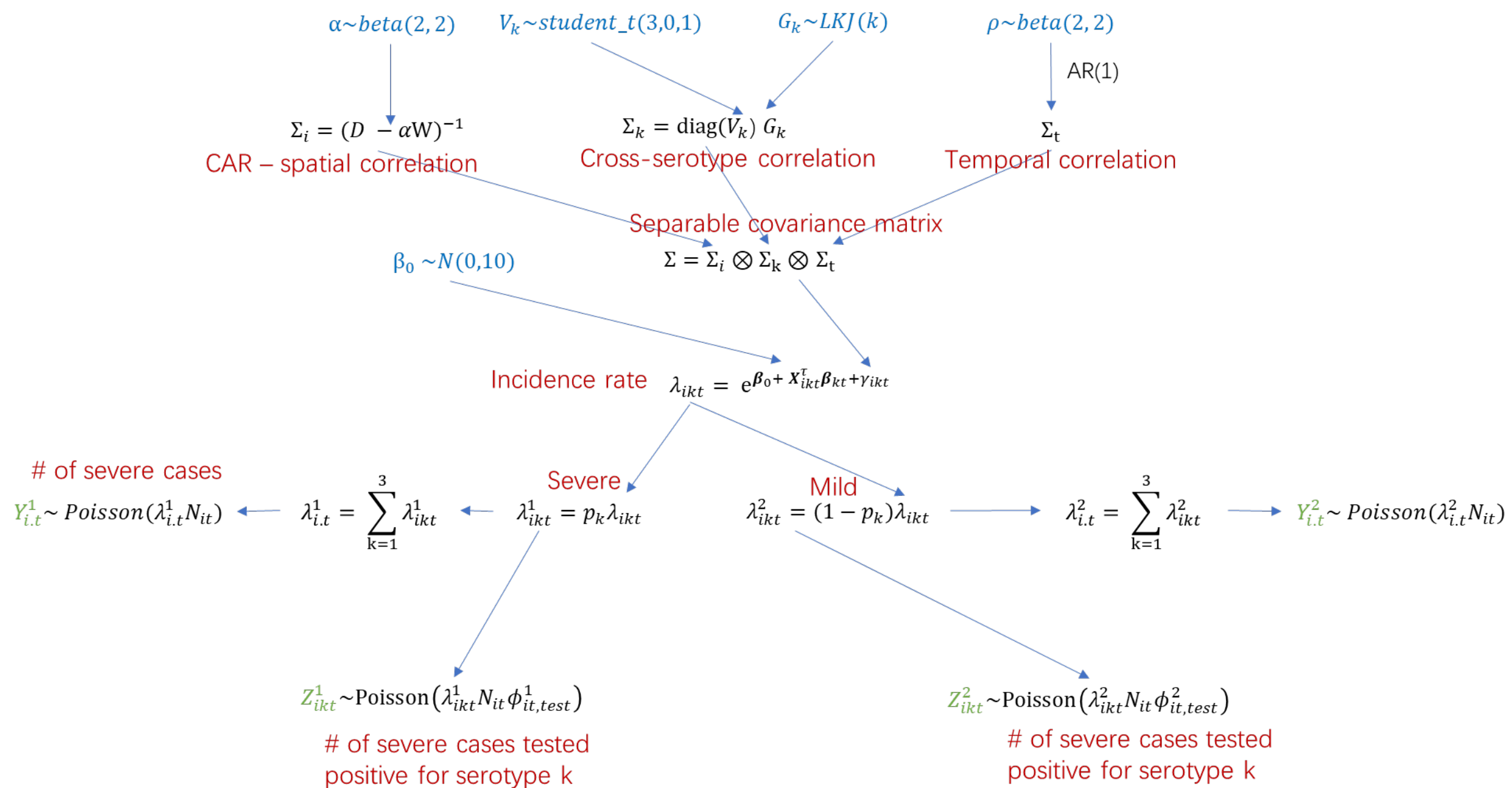

Supplement: S2 Fig — See the main text for the definitions of notations. Priors of the hyperparameters are highlighted in blue, while observed data are highlighted in green. (PDF) [file pcbi.1010575.s003.pdf]

(A) Existing

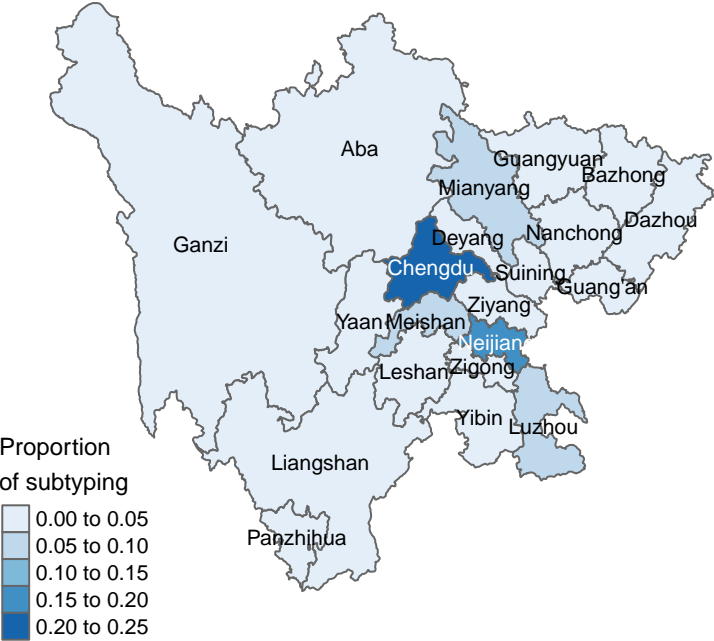

(B) Equal

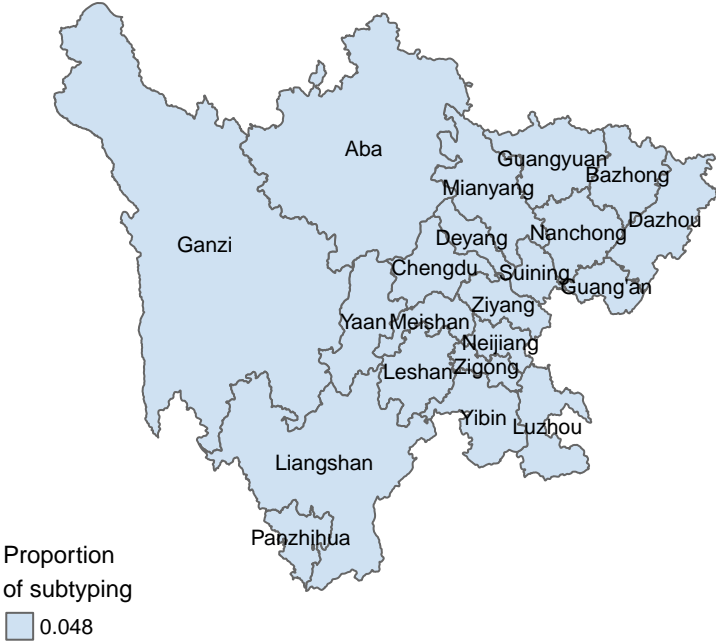

(C) PopSize

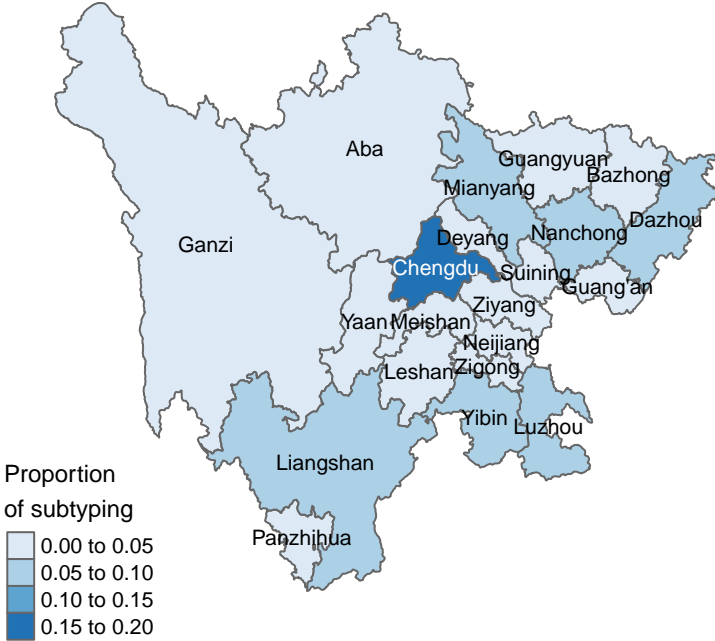

(D) Case

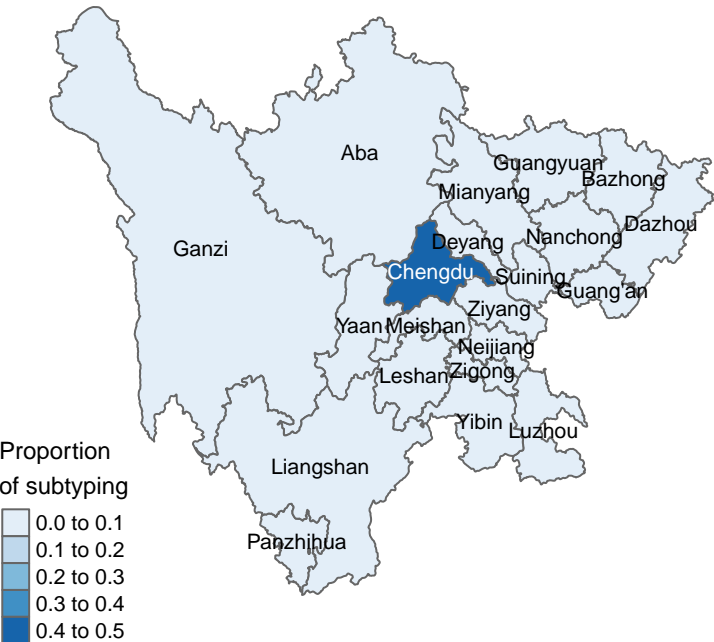

(E) IncRate

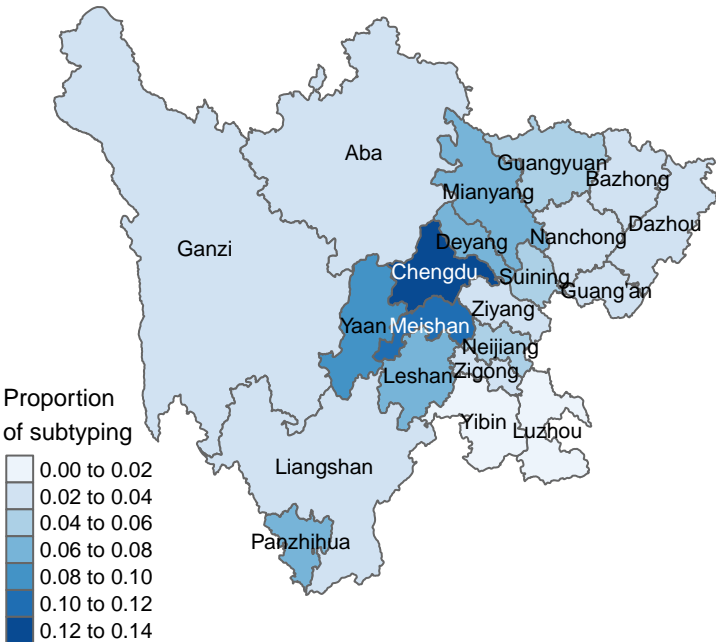

(F) SevereCase

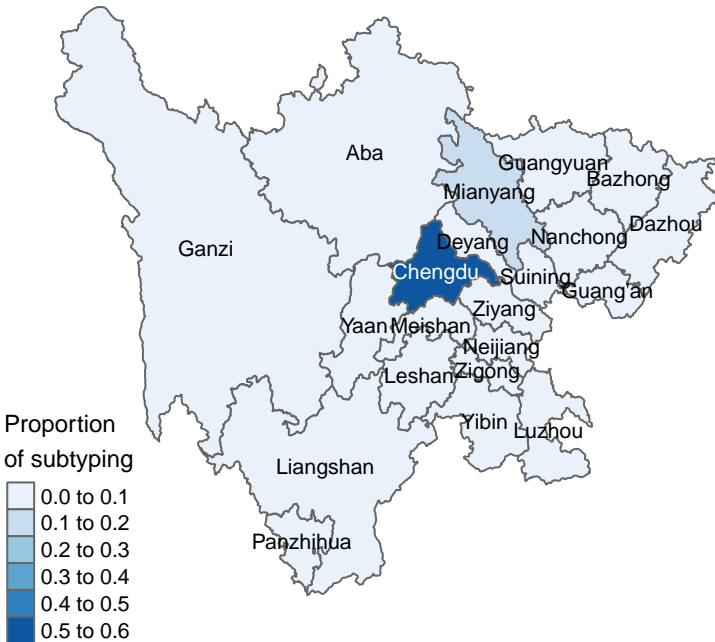

(G) SevereIncRate

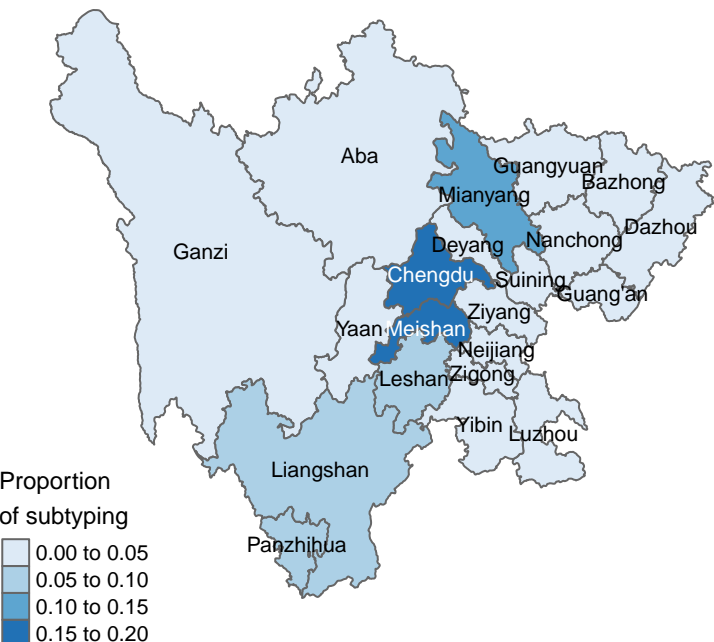

Supplement: S3 Fig — Proportion of typing resources allocate to each location for the archetypal designs: (A) Existing, (B) Equal, (C) PopSize, (D) Case, (E) IncRate, (F) SevereCase, and (G) SevereIncRate. See descriptions of these designs in Section 2.2.7 of the main text. The prefectures are colored by the proportion of serotyping resources allocated to them, with darker colors representing more serotyping resources. The boundaries of the prefectures were obtained from https://gadm.org/download_country.html. (PDF) [file pcbi.1010575.s004.pdf]

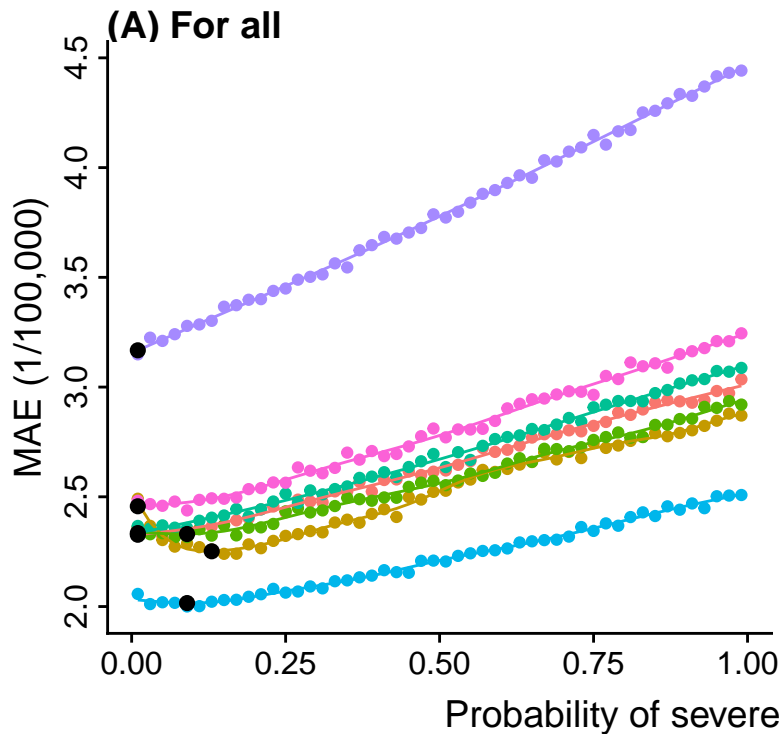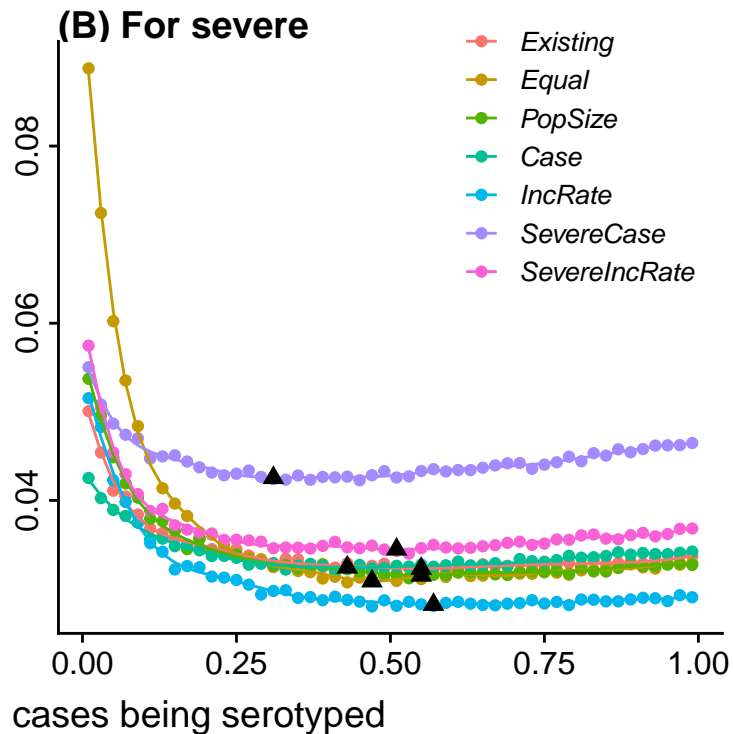

Supplement: S5 Fig — Optimal probability of severe cases being serotyped for each archetypal design minimizing mean absolute errors (MAE) of the estimated serotype-specific incidence rate of (A) all HFMD cases and (B) only severe HFMD cases. Different colors represent different archetypal designs. The colored lines are smoothed by Gaussian Process model. Black dots and triangles represent the optimal probability of severe cases being serotyped for each archetypal design minimizing mean absolute errors (MAE) of the estimated serotype-specific incidence rate of all HFMD cases and only severe HFMD cases, respectively. (PDF) [file pcbi.1010575.s006.pdf]

**(A) For all**

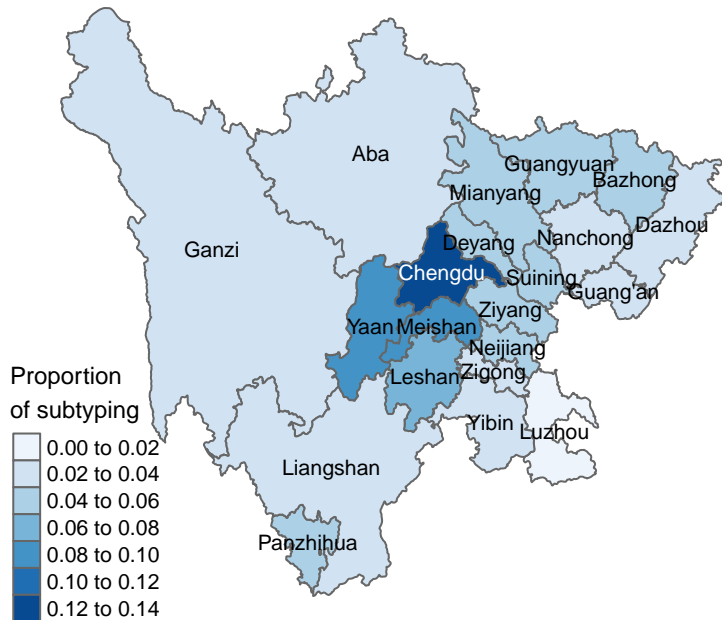

**(B) For severe**

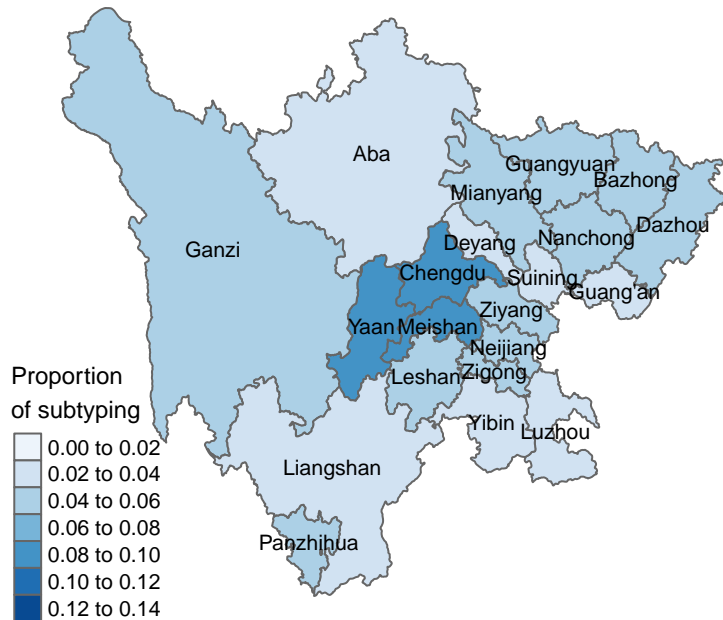

Supplement: S6 Fig — The optimal proportion of subtyping to allocate to each location for minimizing mean absolute error in estimating serotype-specific incidence rate of (A) all cases and (B) severe cases. The boundaries of the prefectures were obtained from https://gadm.org/download_country.html. (PDF) [file pcbi.1010575.s007.pdf]

**(A) For all**

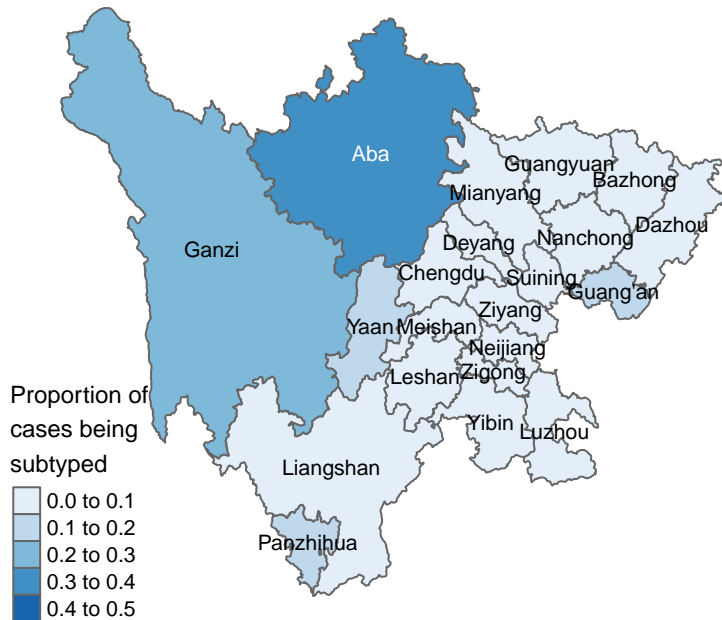

**(B) For severe**

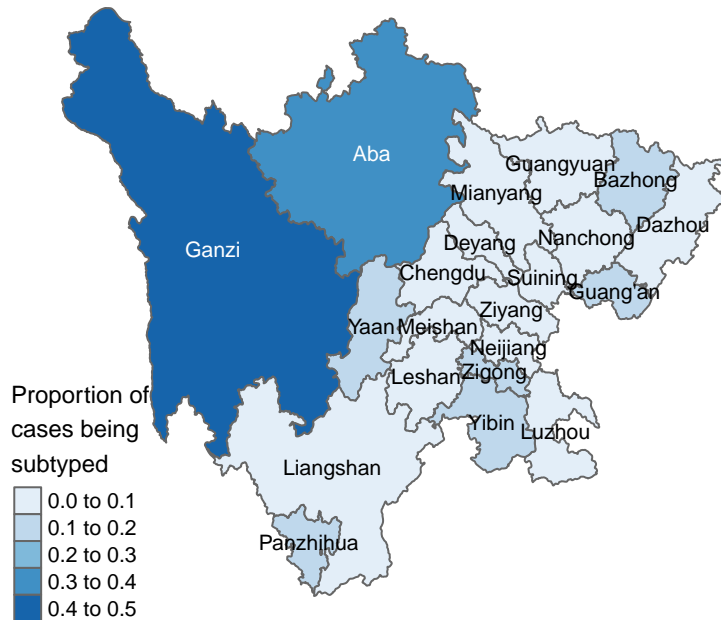

Supplement: S7 Fig — The proportion of cases being subtyped according to the optimal designs that minimize mean absolute error in estimating serotype-specific incidence rate of (A) all cases and (B) severe cases. The boundaries of the prefectures were obtained from https://gadm.org/download_country.html. (PDF) [file pcbi.1010575.s008.pdf]

**(A) For all**

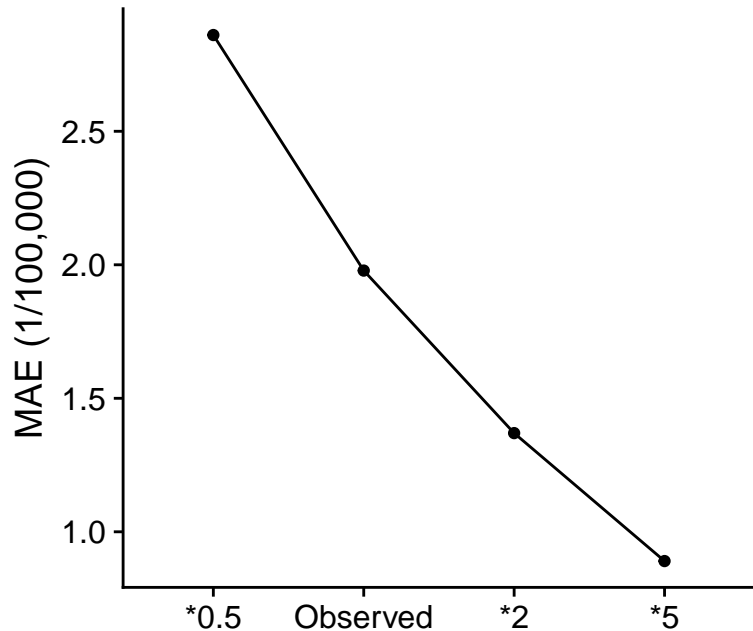

**(B) For severe**

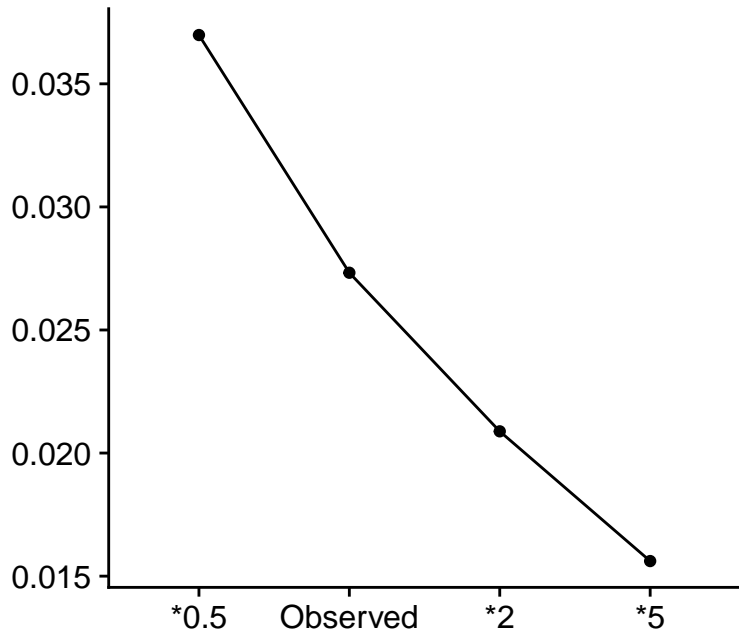

Typing resources

Supplement: S8 Fig — Mean absolute error in estimating serotype-specific incidence rate of (A) all cases and (B) only severe cases when the availability of typing resources changes. (PDF) [file pcbi.1010575.s009.pdf]

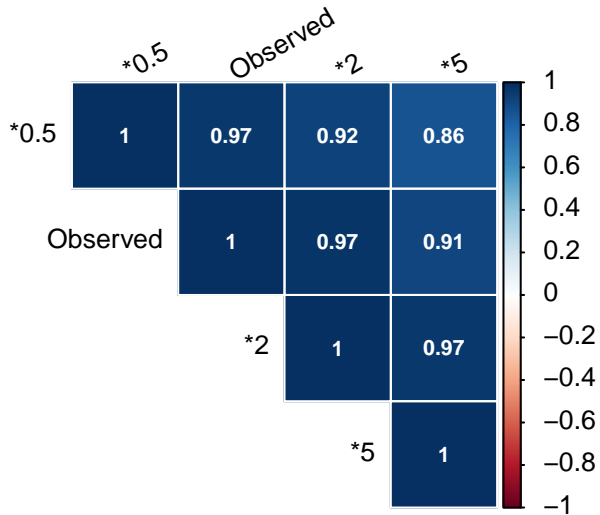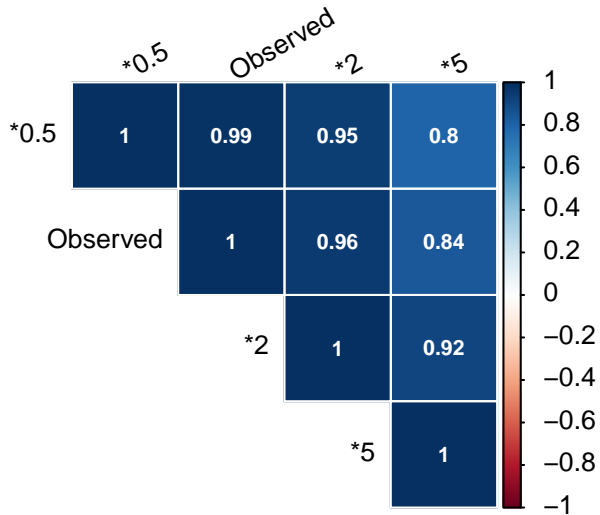

Supplement: S11 Fig — Correlation between the MAEs of estimated serotype-specific incidence rate of (A) all cases and (B) only severe cases. (PDF) [file pcbi.1010575.s012.pdf]
