# Supplementary material for: Optimizing laboratory-based surveillance networks for monitoring multi-genotype or multi-serotype infections
Source: PLoS Comput Biol. 2022 Sep 27;18(9):e1010575. doi: 10.1371/journal.pcbi.1010575 (PMC9543988; doi:10.1371/journal.pcbi.1010575)
Supplement: S4 Fig — Proportion of serotyping resources allocate to each location for the archetypal designs: (A) Existing, (B) Equal, (C) PopSize, (D) Case, (E) IncRate, (F) SevereCase, and (G) SevereIncRate. See descriptions of these designs in Section 2.2.7 of the main text. Each tile represent one location, with the area of the tile proportional to the amount of typing resources allocated to it and the color of the tile representing the annual mean incidence rate of that location. (PDF) [file pcbi.1010575.s005.pdf]

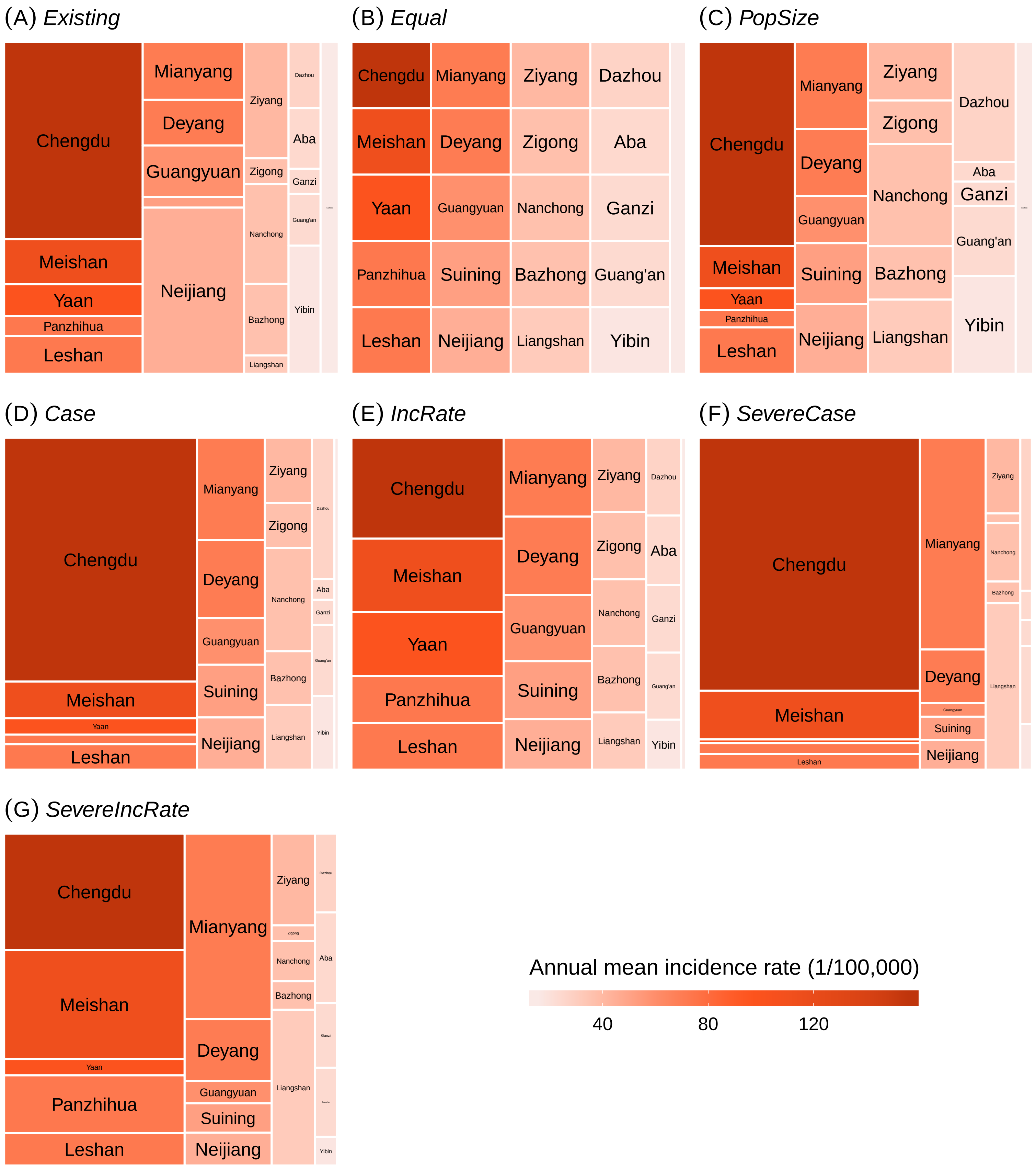
(A) Existing
Chengdu
Meishan
Yaan
Panzhihua
Leshan
Mianyang
Deyang
Guangyuan
Neijiang
Ziyang
Zigong
Nanchong
Bazhong
Liangshan
Dazhou
Aba
Ganzi
Guang'an
Yibin
(B) Equal
Chengdu
Mianyang
Ziyang
Dazhou
Meishan
Deyang
Zigong
Aba
Yaan
Guangyuan
Nanchong
Ganzi
Panzhihua
Suining
Bazhong
Guang'an
Leshan
Neijiang
Liangshan
Yibin
(C) PopSize
Chengdu
Meishan
Yaan
Panzhihua
Leshan
Mianyang
Deyang
Guangyuan
Suining
Neijiang
Ziyang
Zigong
Nanchong
Bazhong
Liangshan
Dazhou
Aba
Ganzi
Guang'an
Yibin
(D) Case
Chengdu
Meishan
Yaan
Leshan
Mianyang
Deyang
Guangyuan
Suining
Neijiang
Ziyang
Zigong
Nanchong
Bazhong
Liangshan
Aba
Ganzi
Yibin
(E) IncRate
Chengdu
Meishan
Yaan
Panzhihua
Leshan
Mianyang
Deyang
Guangyuan
Suining
Neijiang
Ziyang
Zigong
Nanchong
Bazhong
Liangshan
Dazhou
Aba
Ganzi
Guang'an
Yibin
(F) SevereCase
Chengdu
Meishan
Leshan
Mianyang
Deyang
Suining
Neijiang
Ziyang
Nanchong
Bazhong
Liangshan
(G) SevereIncRate
Chengdu
Meishan
Yaan
Panzhihua
Leshan
Mianyang
Deyang
Guangyuan
Suining
Neijiang
Ziyang
Nanchong
Bazhong
Liangshan
Dazhou
Aba
Yibin
Annual mean incidence rate (1/100,000)
40
80
120
