# Supplementary material for: Optimizing laboratory-based surveillance networks for monitoring multi-genotype or multi-serotype infections
Source: PLoS Comput Biol. 2022 Sep 27;18(9):e1010575. doi: 10.1371/journal.pcbi.1010575 (PMC9543988; doi:10.1371/journal.pcbi.1010575)
Supplement: S9 Fig — The optimal proportion of subtyping to allocate to each location for minimizing mean absolute error in estimating serotype-specific incidence rate of all cases when the total amount of subtyping resources is (A) half, (B) two times, or (C) five times that of the observed frequency; and for minimizing mean absolute error in estimating serotype-specific incidence rate of severe cases when the total amount of subtyping resources is (D) half, (E) two times, or (F) five times that of the observed frequency. The boundaries of the prefectures were obtained from https://gadm.org/download_country.html. (PDF) [file pcbi.1010575.s010.pdf]

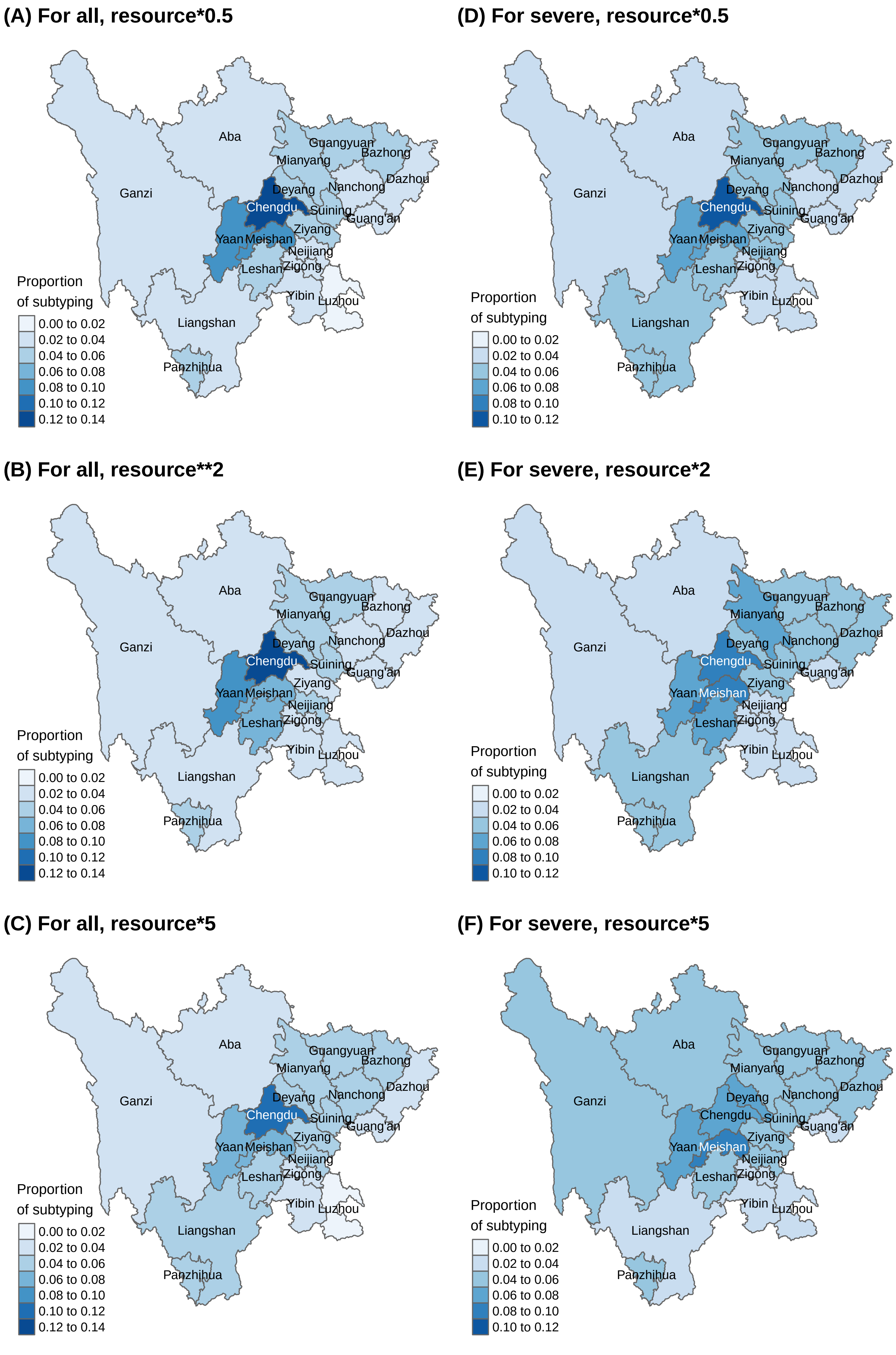
(A) For all, resource*0.5
Aba
Guangyuan
Bazhong
Mianyang
Ganzi
Deyang
Nanchong
Dazhou
Chengdu
Suining
Guang'an
Ziyang
Yaan
Meishan
Neijiang
Leshan
Zigong
Yibin
Luzhou
Liangshan
Panzhihua
Proportion of subtyping
0.00 to 0.02
0.02 to 0.04
0.04 to 0.06
0.06 to 0.08
0.08 to 0.10
0.10 to 0.12
0.12 to 0.14
(D) For severe, resource*0.5
Proportion of subtyping
0.00 to 0.02
0.02 to 0.04
0.04 to 0.06
0.06 to 0.08
0.08 to 0.10
0.10 to 0.12
(B) For all, resource**2
Proportion of subtyping
0.00 to 0.02
0.02 to 0.04
0.04 to 0.06
0.06 to 0.08
0.08 to 0.10
0.10 to 0.12
0.12 to 0.14
(E) For severe, resource*2
Proportion of subtyping
0.00 to 0.02
0.02 to 0.04
0.04 to 0.06
0.06 to 0.08
0.08 to 0.10
0.10 to 0.12
(C) For all, resource*5
Proportion of subtyping
0.00 to 0.02
0.02 to 0.04
0.04 to 0.06
0.06 to 0.08
0.08 to 0.10
0.10 to 0.12
0.12 to 0.14
(F) For severe, resource*5
Proportion of subtyping
0.00 to 0.02
0.02 to 0.04
0.04 to 0.06
0.06 to 0.08
0.08 to 0.10
0.10 to 0.12
