# Supplementary material for: Optimizing laboratory-based surveillance networks for monitoring multi-genotype or multi-serotype infections
Source: PLoS Comput Biol. 2022 Sep 27;18(9):e1010575. doi: 10.1371/journal.pcbi.1010575 (PMC9543988; doi:10.1371/journal.pcbi.1010575)
Supplement: S10 Fig — Scatterplots of annual mean incidence rate and the proportion of typing resources allocating to each location under the archetypal design IncRate (black dots) and the Optimal designs for minimizing the MAE of estimated serotype-incidence rate of all HFMD cases (blue triangles) when the available typing resources is (A) halved, (B) doubled, and (C) quintupled; and the Optimal designs for minimizing the MAE of estimated serotype-incidence rate of severe HFMD cases (blue squares) when the available typing resources is (D) halved, (E) doubled, and (F) quintupled. (PDF) [file pcbi.1010575.s011.pdf]

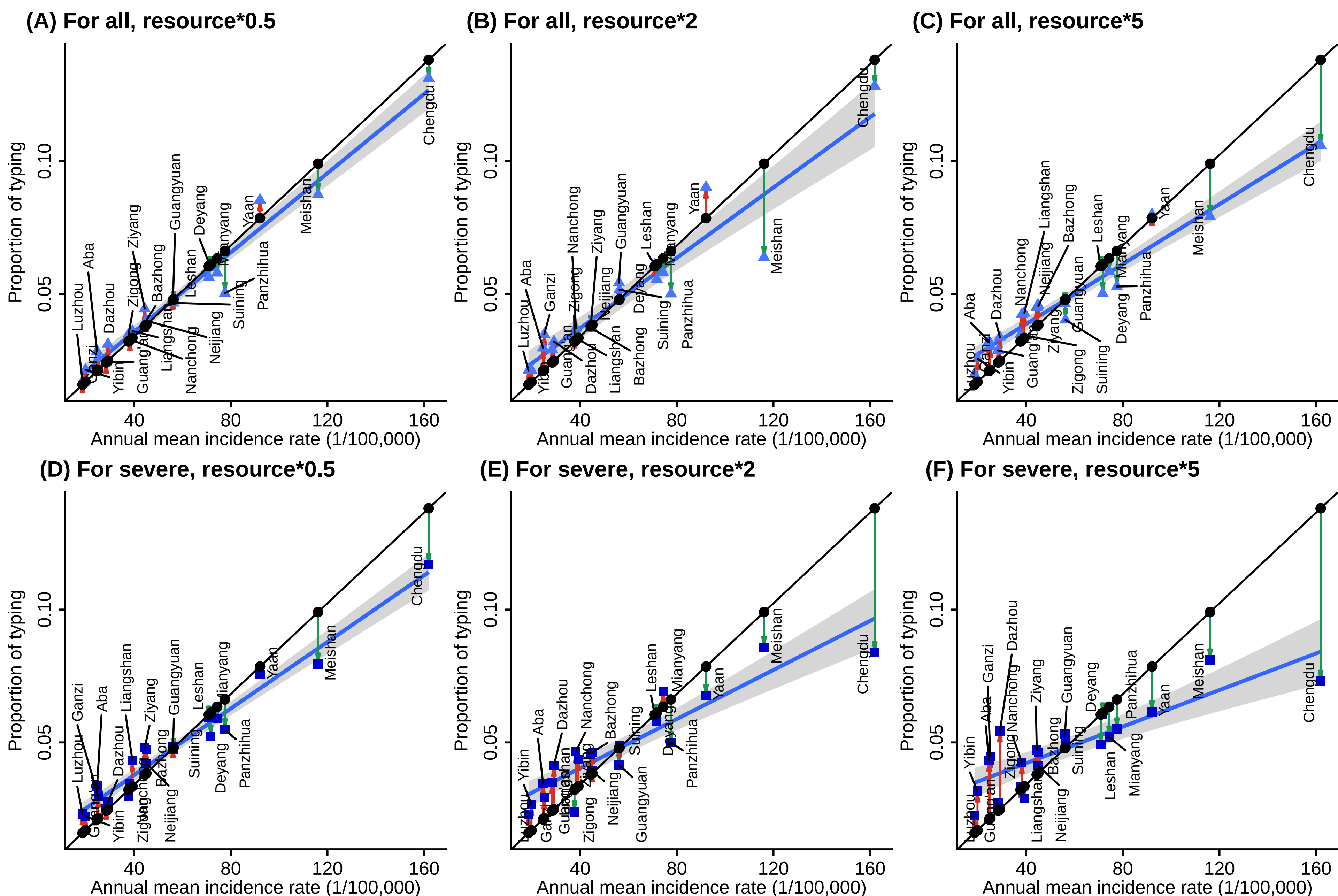

(A) For all, resource*0.5
(B) For all, resource*2
(C) For all, resource*5
(D) For severe, resource*0.5
(E) For severe, resource*2
(F) For severe, resource*5
Proportion of typing
Annual mean incidence rate (1/100,000)
0.05
0.10
40
80
120
160
Chengdu
Meishan
Yaan
Panzhihua
Mianyang
Deyang
Leshan
Guangyuan
Suining
Neijiang
Nanchong
Liangshan
Bazhong
Ziyang
Zigong
Dazhou
Aba
Ganzi
Yibin
Luzhou
Guang'an
