# Supplementary material for: Optimizing laboratory-based surveillance networks for monitoring multi-genotype or multi-serotype infections
Source: PLoS Comput Biol. 2022 Sep 27;18(9):e1010575. doi: 10.1371/journal.pcbi.1010575 (PMC9543988; doi:10.1371/journal.pcbi.1010575)
Supplement: Attachment — Submitted filename: HFMD_lab_surveillance_response_letter_R1.docx [file pcbi.1010575.s013.docx]

**Reviewer's Comments**

**Reviewer #1**

General: This is a Research Article submission by Cheng and colleagues focused on development of an analytic framework to improve decisions on sampling for infectious disease surveillance with the case example of Hand-foot-and-mouth disease in China. They model where to allocate a fixed number of PCR assays that identify viral etiology (CA-V16, EV-A71, or other) amongst N=21 locations with a goal of optimizing serotype specific case incidence. Overall this is an interesting analysis. My main concern is whether the data can truly support the authors’ goals.

Major comments:
-The goal of the analysis is to compare a model-driven sampling scheme to current practice (archetypal). Therefore a reference standard is needed to identify the “true” serotype specific incidence, by which to compare these approaches. Ideally, this would be high resolution sampling of all locations. However, the data from almost all locations except Chengdu appears quite limited (Figure 2D). Therefore, even with the approach done by the authors with resampling, I worry the data is limited to make an informed comparison. Would welcome clarification and input from the authors, and additional description of the available data.

**We fully agree with the reviewer that high resolution sampling across space and time would provide the best basis for assessing the performance of alternative surveillance designs—and would in fact be, in and of itself, *the* *optimal* *sampling design* in the absence of logistical, resource, and other constraints. However, real-world limitations of data collection necessitate alternative approaches to comparison. Here, we simulate true serotype specific incidences under uncertainty in order to infer posterior distributions of the underlying incidences from observed data. We used a model that was previously validated for similarly sparse, multi-pathogen data in China [1]; the framework allows borrowing of information across space, time, and pathogen type to compensate for sparse observations In the present work, we validated the optimized design against data from 2015, which were withheld during the optimization process. The results of the disease and surveillance models were consistent with observed data over this period.**

**In the revised Discussion (L587, the tracked change version), we added mention of the value of comprehensive, cross-sectional sampling to establish data for validation of disease and surveillance models and the resulting optimal surveillance design:**

**“Periodic intensive, cross-sectional sampling may also help to validate and fine-tune the design optimization process by providing high-resolution, high-confidence estimates of incidence rates.”**

Minor comments:
-I am not clear how the DIOS framework is particularly unique beyond an iterative algorithm for this case example.

**We appreciate the comment. What is unique about the DIOS framework is its systematic approach to data-driven surveillance design. The framework fully identifies and specifies core surveillance components (e.g., probabilistic model(s) of disease conditioned on observed data; surveillance information abstracted from underlying epidemiological processes as a function of design factors; etc.) and procedures (e.g., simulation-optimization) that can be applied to a broad range of objectives and contexts [2]. Indeed, this broad applicability may render individual case studies such as the one presented in this paper somewhat less ‘unique’ in that each will represent a synthesis of general simulation optimization principles with process models suitable to the context of the study, many of which may be derived from previously published work. One of our goals for this paper was to illustrate an extension of DIOS to a previously unexplored problem with practical relevance—multi-type pathogen surveillance under common constraints. Our hope is that this and similar case studies demonstrating its possibilities may inspire readers to adapt the framework to their own ends.**

-Could the authors clarify the “Realizations” from equation on line 269 and why there are 80?

**To propagate uncertainties in the underlying serotype-specific HFMD incidence rates, we draw multiple samples from their estimated posterior distributions, which we call realizations. To clarify the terminology for readers, we now define the term in the revised manuscript on L270:**

**“…across locations, time, serotypes, and realizations (i.e., samples from the posterior distribution) of the disease system model”**

**We have added a brief explanation for the selection of 80 realizations on L275-276:**

**“…(R = 80, selected to ensure convergence of the estimated MAE across model runs)…”**

-There appears to be the assumption that the number of samples is fixed rather than variable, which could be further explored.

**We appreciate this comment. We did in fact vary the total number of samples and examined how available resources (i.e., the number of cases selected for subtyping) can affect surveillance performance and the optimal design. We presented these results in the original manuscript section *3.3 Sensitivity of selected designs to the total number of samples for subtyping*. To summarize those findings, as more cases were sampled for subtyping, surveillance performance (i.e., mean absolute error of the estimated serotype-specific incidence rates) improved substantially; the optimal allocation of tests across sites changed modestly (as determined by the minimum average site-level error in serotype-specific incidence rates); and optimal allocation of tests to minimize average site-level errors in serotype-specific incidence rates among severe cases changed substantially, for reasons we discuss in the main text (see L533-537). If we can clarify these findings further for readers, we would be glad to do so.**

-How do the authors deal with uncertainty?

**We view robust treatment of uncertainty as essential to the simulation-optimization approach of DIOS. The primary method by which we incorporate uncertainty within DIOS is by generating realizations of disease data and surveillance data from probabilistic models of the underlying processes. We describe this in the methods (L325-331):**

**“To ensure that our optimized surveillance scenarios account for uncertainty in the observation process and parameter estimates, after fitting the disease process and observation model to data from 2009-2014, R sets (R = 80) of serotype-specific incidence rates (**$\boldsymbol{\lambda}_{\boldsymbol{ikt}}^{\boldsymbol{(r)}}$**) and parameter values (including** $\boldsymbol{\beta}_{\boldsymbol{0}}$ **and hyperparameters of** $\boldsymbol{\Sigma}$**) were drawn from the joint posterior distributions. The sampled parameter values were used by the surveillance model (section *2.2.5*) to simulate** $\boldsymbol{Z}_{\boldsymbol{ikt|\theta}}^{\boldsymbol{(s)}}$ **and to estimate** ${\hat{\boldsymbol{\lambda}}}_{\boldsymbol{ikt|\theta}}^{\boldsymbol{(r)}}$ **and** ${\hat{\boldsymbol{p}}}_{\boldsymbol{k|\theta}}^{\boldsymbol{(r)}}$ **under different surveillance designs.”**

-The authors mention additional dimensions of time and serotype, is varying these for optimization explored? Would be reasonable to do only location, just curious.

**Though we have limited ourselves to addressing a *spatial* design problem in the current manuscript, the DIOS framework and methods presented here can indeed be applied to optimally allocating typing resources across time, for instance across seasons. With respect to targeting specific serotypes, one might use historic serotype information to inform test distribution over time, space, or across certain subpopulations. While these analytical areas fall outside the scope of the present manuscript, we share the reviewer’s curiosity surrounding such applications, and hope to pursue them in future work.**

-Methods section is somewhat hard to follow.

**We agree that the methods section is dense and complicated, which was necessary to provide sufficient detail to facilitate replication of the procedures by others. In the revision, we have attempted to clarify several sections within the methods and we would gladly strengthen other areas that reviewers/editors recommend for further clarification.**

____________________________________________________________

**Reviewer #2**

Thank you for the chance to review the research article submission "Optimizing laboratory-based surveillance networks for monitoring multi-genotype or multi-serotype infections". This paper is a clear, well written, original, innovative and potentially rather important contribution to the literature on disease surveillance system design, evaluation and optimization. While the paper may appear to overlap in-part with the prior publication by Cheng et al in PLOS Comp Biol from 2020, https://journals.plos.org/ploscompbiol/article?id=10.1371/journal.pcbi.1008477, the current submission presents a very nice methodological advance in this area.

A few issues with editing and clarity are outlined below (by page and line number),

Page 5, Line 98-99: re. "and only 2-3 influenza cases are required to be typed..." Is this a typo, and should be indicating 2-3% of influenza cases, or does CDC and APHL actually say "2-3 influenza cases", and if so, I gather they also stipulate some denominator, possibly such as 2-3 cases per specified geography or time period or population.

**Our thanks for spotting this; we had indeed missed the denominator in the original sentence. In the revision, we corrected the sentence to read：**

**“…only 9 influenza cases per participating laboratory are required to be characterized every other week across the United States to evaluate whether circulating influenza viruses are sufficiently similar genetically and/or antigenically to those that are included in current influenza vaccines”**

P6 L110-118: re. "biases arising from sampling clinical cases for subtyping..." This language seems to imply that sampling follows a scheme that is intentionally designed in most cases, but I worry that it is the absence of design that is actually more typical. The academic and engineering perspective should clearly be informing hospital, clinical, laboratory and public health systems and practices. I just wonder if the assumptions in this paper are too far from reality, and a more basic and simple set of guidelines need to be met first by public health surveillance authorities.

**We wholehearted agree with the reviewer that design of the real-world surveillance systems is typically *ad hoc* and not informed by quantitative analyses. We are working closely with collaborators at China Centers for Disease Control and Prevention in the hopes that DIOS may gradually shift practice towards the development and implementation of data-driven design schema. We also understand the many barriers to the adoption of optimization frameworks within real-world public health systems. It is for this reason that we compared the performance of numerically optimized designs with a variety of ‘rule of thumb’ designs (e.g., resource allocation in accordance with average historical incidence). These results clarify what the optimization procedure is selecting for, and provide practitioners with potentially actionable insights into system performance across a range of designs. In the revision, we have updated this section (at L113) to lay out the full set of issues that *ad hoc* designs raise:**

**“Such imbalanced sampling regimes, often arising from practical clinical considerations, can substantially impact estimates of genotype-, serotype-, or other subtype-specific epidemiologic parameters…”**

P7 L138-143: re. "Applying DIOS involves specifying surveillance objectives..." Again, this may be assuming that surveillance systems are being developed, implemented, and are operating under more optimistic conditions then they actually are. While it is not the place for this paper to necessarily address or try to resolve the deficiencies of public health systems, it might be useful to recognize how far from optimal such practices are. To meaningfully run DIOS in most public health settings would likely require a major investment and a realignment in approach.

**We agree, and have found that revealing how far from optimal the current practice is can motivate important discussion and some progress (such as in our engagement with colleagues at China Centers for Disease Control and Prevention). For the optimization problem we address in the present manuscript, we examined the performance of an existing surveillance system and found (Figure 5) that it performed significantly worse than our optimal and than the best rule-of-thumb designs.**

P10 L172-174: very true re. dynamics, and that it should represent known biases due to subtype interaction, but also possibly unrecognized interactions between variants/subtypes that that are not (yet) known.

**We agree that this is an important—but challenging—application of the framework. The disease system model is most likely to be developed based on what we already know about the disease. When the underlying mechanism is not clear, best practice would likely involve ensemble models representing multiple credible descriptions of the disease (and/or surveillance) processes to represent this uncertainty. We discuss this in the present manuscript (L583-586):**

**“Expanding on the use of a single disease system model here, multiple models with different structures—e.g., hierarchical models with different covariance structures, machine learning algorithms, and mechanistical models—and different parameter values or covariates could be run in an ensemble to better represent uncertainty in the underlying epidemiologic processes.”**

**and in our previous paper [2] (which we cite in the present manuscript on L138, among other places):**

**“When a disease system is poorly understood, as in the case of a novel emerging disease (e.g., Coronavirus Disease 2019), optimal surveillance system designs are likely to be subject to considerable epistemic uncertainty. This uncertainty can be represented within the DIOS framework by using an ensemble of plausible disease system models to simulate epidemiologic states during optimization. The resulting variance in surveillance performance (i.e., objective function values) across the design space may result in identification of a large set of designs that are not significantly Pareto dominated on one or more objectives, but it may still be possible to exclude large under-performing regions of design space. As knowledge of the target disease evolves, the ensemble of disease system models can be refined by updating weights for different model structures or adjusting parameter distributions, and optimal designs can be re-evaluated.”**

P26 L457 and L460: possibly redundant, should it just be "GA" instead of "GA algorithm" here?

**Correct. Thanks for pointing it out. We deleted “algorithm” in the revision at L461 and L464.**

**References cited in the *Response to reviews***

1. Tang X, Yang Y, Yu HJ, Liao QH, Bliznyuk N. A spatio-temporal modeling framework for surveillance data of multiple infectious pathogens with small laboratory validation sets. Journal of the American Statistical Association. 2019 Oct 2;114(528):1561-73.

2. Cheng Q, Collender PA, Heaney AK, Li X, Dasan R, Li C, Lewnard JA, Zelner JL, Liang S, Chang HH, Waller LA. The DIOS framework for optimizing infectious disease surveillance: Numerical methods for simulation and multi-objective optimization of surveillance network architectures. PLoS computational biology. 2020 Dec 4;16(12):e1008477.
